# Fragility Fractures of the Pelvic Ring: Analysis of Epidemiology, Treatment Concepts, and Surgical Strategies from the Registry of the German Pelvic Multicenter Study Group

**DOI:** 10.3390/jcm14092935

**Published:** 2025-04-24

**Authors:** David B. Osche, Emmanouil Liodakis, Stefan Huber, Tim Pohlemann, Christian Kleber, Steven C. Herath, Andreas Höch

**Affiliations:** 1 Department of Trauma, Hand and Reconstructive Surgery, Saarland University Medical Center, 66421 Homburg, Germany; 2 AUC—Academy for Trauma Surgery, 80538 Munich, Germany; 3 Department of Orthopedics, Trauma and Plastic Surgery, Leipzig University Medical Center, 04103 Leipzig, Germany; 4 Department of Trauma and Reconstructive Surgery, BG Hospital Tübingen, 72076 Tübingen, Germany

**Keywords:** fragility fractures of the pelvis, pelvic ring stabilization, fracture distribution, geriatric pelvic ring fracture, surgical treatment, non-operative treatment

## Abstract

**Background:** Fragility fractures of the pelvic ring (FFPs) represent a fast-growing entity in geriatric traumatology with increasing incidence worldwide. This study aimed to analyze the epidemiology, treatment concepts, and surgical strategies for FFPs based on data collected by the German Pelvic Multicenter Study Group documented in the German Pelvic Fracture Registry. It is the largest cohort study of its kind. **Methods:** This retrospective cohort study included patients aged 65 years or older after FFPs, as classified according to the Rommens and Hofmann classification. Data were collected from July 2018 onward and analyzed for demographics; fracture classifications; treatment modalities (operative vs. non-operative); and details of surgery, including timing and choice of implants. Patients after high-energy trauma were excluded. Statistical analyses included descriptive metrics and subgroup comparisons. **Results:** Among 1242 patients (84% female; median age 83.4 years), FFP Type II was the most common fracture type (50.8%), followed by Type IV (21.1%). Non-operative management was employed in 68.8% of cases, while 30.9% underwent surgery. Surgical intervention was more frequent in higher-grade FFPs (e.g., 72.1% in Type IV). The most common surgical technique for the posterior pelvic ring was percutaneous screw fixation (61.3%), with navigation used in 47.4% of cases. **Conclusions:** This study highlights the variability in treatment strategies for FFPs, with conservative management predominating in lower-grade fractures and surgical approaches increasingly utilized for more complex cases. The findings underscore the need for standardized, evidence-based guidelines and further research to optimize treatment and long-term outcomes for geriatric patients with FFPs.

## 1. Introduction

Fragility fractures of the pelvic ring (FFPs), similar to proximal femoral fractures in the elderly, represent a severe type of injury. The incidence of FFPs has risen steadily in recent years, overtaking high-energy pelvic trauma primarily affecting young polytraumatized patients and representing the third most common injury in the geriatric population in Germany [1,2].

Taking into account a different injury mechanism, Rommens, Hofmann et al. [3] in 2013 proposed a specific CT-based pelvic fracture classification for FFPs based on a primary loss of bony stability. Before, this was not well represented in the existing pelvic ring classifications, which focus on the result of high energy transfer in healthy bone. The FFP classification, based on a monocentric patient cohort, provides therapeutic recommendations for specific fracture types drawn from clinical experience. However, even this system does not offer definitive treatment guidelines for geriatric pelvic ring fractures [4].

Therapeutic decisions in elderly patients with pelvic fractures are influenced not only by the fracture classification but also by factors such as pain levels and mobility [5,6]. Furthermore, the past decade has witnessed significant advancements in surgical techniques and treatment strategies, particularly the adoption of minimally invasive approaches [7]. Despite these developments, the evidence base for the optimal management of these fractures remains limited, and guidelines are missing [8,9,10].

This study aims to analyze registry data to provide insights into the current epidemiology, treatment concepts, and surgical strategies for FFPs in order to reveal recent best practice for the establishment of a national guideline. Additionally, the findings are compared to the initial recommendations proposed by Rommens et al. [4] to evaluate contemporary practices in the management of these injuries:

- Are FFP Type I injuries predominantly treated non-operatively?
- Are less invasive surgical techniques already applied?
- How common is the combined fixation of both the posterior and anterior pelvic ring in FFPs?

## 2. Materials and Methods

This retrospective cohort study utilized data from the German Pelvic Fracture Registry, which is part of the TraumaRegister DGU^®^ of the German Trauma Society (DGU). The database is hosted and managed by the AUC—Academy for Trauma Surgery.

A total of 40 trauma centers across Germany contribute to the registry by entering pseudonymized data from patients with pelvic injuries, contingent upon obtaining patient consent. Data collection is conducted in compliance with the General Data Protection Regulation (GDPR) and is approved by the respective local ethics committees in each federal state.

Data retrieval from the registry was performed on 16 September 2024. For the analysis, only cases entered into the database from July 2018 onward were included, as this marks the introduction of the FFP classification in the registry. Of all the trauma centers contributing to the registry, for this study, consecutive data from 21 German and 1 Austrian trauma center were included.

**Figure 1:**
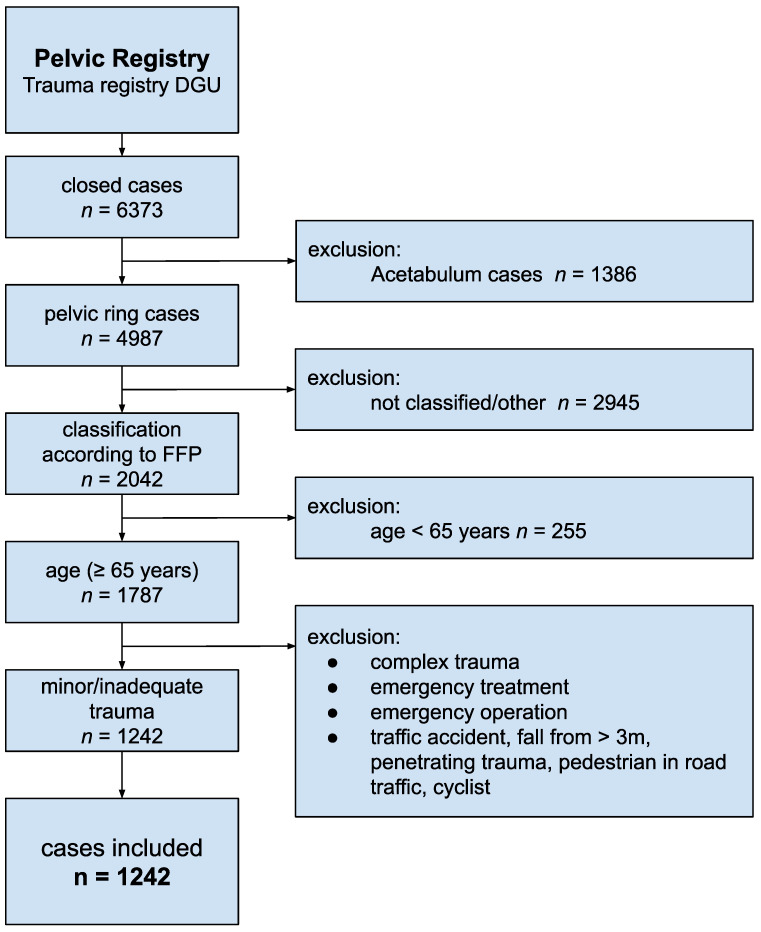
Inclusion criteria.

All patients aged 65 years or older who were classified according to the FFP criteria (low-energy/non-existing trauma) were included in the study. Additionally, patients needed to have sustained a minor trauma, such as a fall from a standing height, or an unspecified trauma. Cases involving high-impact trauma (e.g., falls from significant heights, pedestrian accidents, bicycle accidents, or car accidents) and their associated consequences (e.g., shock room admission, emergency treatment) were excluded from the study population. Additional exclusion criteria are detailed in the flowchart presented in Figure 1.

The parameters evaluated in this study included patient demographics such as age and sex as well as clinical factors like the FFP classification and the treatment modality, categorized as either operative or non-operative. Time-related variables were also analyzed, including the interval between accident and operation and the time from admission to the emergency department to operation. Additionally, surgical details were assessed, focusing on the surgical techniques employed as well as the selection of implants.

### 2.1. Operative Treatment

The evaluation of operative therapy was based on the anatomical localization, the type of procedure performed, and the implants used. To clarify the surgical approaches, this study distinguishes between the anterior and posterior pelvic ring. Within the pelvic registry, the anterior pelvic ring encompasses the pubic symphysis and the os pubis. Notably, no cases in the FFP cohort required symphyseal stabilization; therefore, the term “anterior pelvic ring” exclusively refers to the os pubis in this analysis.

The term “posterior pelvic ring” in this study includes the ilium proximal to the hip joint, the sacroiliac (SI) joint, and the sacrum, as categorized within the registry.

### 2.2. Statistical Analysis

Statistical analysis was conducted using R R 4.4.0 (The R Foundation for Statistical Computing, Vienna, Austria). Descriptive statistics summarized demographics, fracture classifications, treatment modalities, and time intervals, such as the duration from the accident to surgery. Results were expressed as absolute numbers, percentages, means, and standard deviations for normally distributed data and medians and IQRs for non-normally distributed data.

Analyzing the data, we assessed differences in surgical techniques, implant choices, and treatment outcomes, stratified by FFP classification. Treatment distributions were analyzed to highlight trends and ensure data reliability.

## 3. Results

The study cohort comprised 1242 patients who were diagnosed with an FFP, of whom 1039 (84%) were female and 203 (16%) were male. The median age was 84 years (IQR 79–88). According to the American Society of Anesthesiologists (ASA) classification, 5.4% of the patients were categorized as healthy (ASA 1), 35.4% as moderately ill (ASA 2), 41.4% as critically ill (ASA 3), and 1.1% as having a life-threatening condition (ASA 4). However, 16.7% of the patients in the collective were not classified according to the ASA classification.

### 3.1. FFP Classification

The distribution of fracture types according to the FFP classification highlights that FFP Type II fractures were the most common, accounting for 50.8% of cases, particularly in subcategories IIb (31.7%) and IIc (13.3%), which represent moderate to severe posterior pelvic ring instability. FFP Type IV fractures followed with 21.1%, with subcategory IVb (15.4%) contributing significantly to the complex injuries. FFP Type I fractures comprised 20.8% of cases, while FFP Type III was the least frequent at 7.3%. This distribution reflects the varying severity of fragility fractures within the studied population. A detailed breakdown is summarized in Table 1.

### 3.2. Therapy

Among the 1242 patients analyzed, 854 patients (68.8%) received non-operative treatment, while 384 patients (30.9%) underwent surgical intervention. For four patients (0.3%), the type of therapy administered was not specified. Surgical procedures were most commonly performed in patients with injuries classified as FFP Type IV (72.1%) and FFP Type III (61.5%). FFP Type I injuries were predominantly managed non-operatively (97.3%), and FFP Type II injuries were largely treated non-operatively (78.8%).

**Figure 2:**
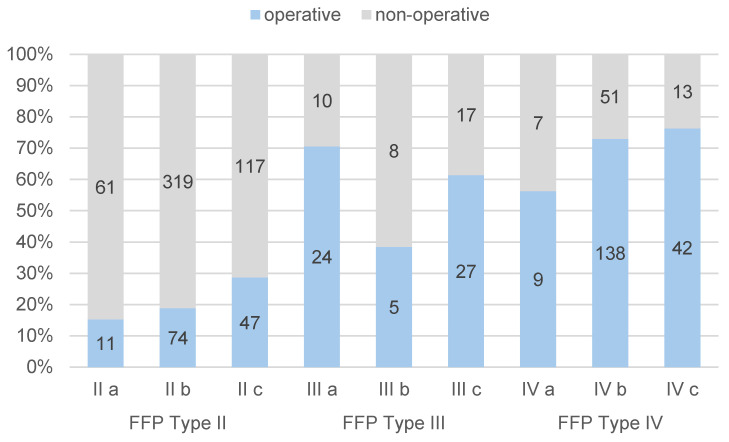
Treatment type based on FFP classification (operative vs. non-operative). The numbers on the bars are absolute values.

The relative frequencies of surgical treatment across FFP subgroups are detailed in Figure 2, highlighting the treatment patterns for different fracture types.

### 3.3. Timing of Surgery

Data on the time interval between the accident or onset of symptoms and surgical intervention was available for 374 of the 384 patients who underwent surgery (97.4%). The median duration from the accident to surgery was 9 days (IQR 4.25–17). The median duration from admission to surgery was 5.5 days (IQR 3–9).

### 3.4. Choice of Implants

There are multiple implants as well as combinations of implants used in the surgical treatment of FFPs. An overview is shown in Table 2.

#### 3.4.1. Anterior Pelvic Ring

For the stabilization of the anterior pelvic ring (n = 139), the percutaneous screw technique was the most commonly employed method, used in 75 patients (54.0%). This approach was predominantly performed without navigation (n = 68, 90.7%) and only rarely with navigation (n = 7, 9.3%). It must be noted that the term “navigation” can either describe the ISO-C-3D navigated placement of screws or whether a CT scan was obtained to verify the correct placement of a k-wire for a cannulated screw, since there is no further discrimination possible in the registry. The supraacetabular external fixator was utilized in 33 patients (23.7%). Less frequently used techniques included internal fixators (n = 10, 7.2%) and open reduction with plate osteosynthesis (n = 21, 15.1%).

#### 3.4.2. Posterior Pelvic Ring

For stabilizing iliac fractures (n = 33), screw osteosynthesis was the most commonly used technique, applied in 14 patients (42.4%). Among these, non-navigated screws were more frequently used (n = 12, 86%). Plate osteosynthesis was performed in 11 patients (33.3%). A combination of screw and plate osteosynthesis was performed in five cases (15.2%). Additionally, three patients (9.1%) underwent stabilization procedures that were not further specified.

The most frequently utilized technique for fixation of the SI joint and sacral fractures (n= 272) was percutaneous screw osteosynthesis, performed in 171 patients (63%). This approach also allowed for differentiation between navigated (n = 82, 47%) and non-navigated (n = 91, 53%) implantation. In 57 cases (33%), the specific treatment plan was not further detailed. Open stabilization was performed in 41 patients (24%) in this region. Following open reduction, a spinopelvic fixation was performed in 28 cases (68%).

In summary, stabilization of the posterior pelvic ring (n = 304) was primarily achieved using closed reduction and percutaneous screw osteosynthesis, performed in 187 cases (62%). Among these, navigation techniques were utilized in 84 cases (45%).

### 3.5. Stabilization Concepts

There are different surgical options for the stabilization of FFPs. The distribution is shown in Table 3.

#### 3.5.1. Isolated Stabilization of the Anterior Pelvic Ring

Of the 384 patients that were treated operatively, isolated stabilization of the anterior pelvic ring was rare and performed in only eight cases (2.1%). Of these, five patients (62.5%) had FFP Type I injuries, two (25%) had FFP Type II injuries, and one (12.5%) had an FFP Type IV injury.

#### 3.5.2. Isolated Stabilization of the Posterior Pelvic Ring

Isolated stabilization of the posterior pelvic ring was far more common in the collective, performed in 248 cases (64.6%). This operative method was used in 50% of both operated FFP Type II (n = 66) and III (n = 28) injuries and in 80.4% (n = 152) of operated FFP Type IV injuries.

#### 3.5.3. Combined Anterior and Posterior Stabilization of the Pelvic Ring (“360°”)

Combined stabilization was utilized in 33.1% (n = 127) of all operated FFPs (n = 384). Additionally, 47.7% of all operated FFP Type II injuries and 50.0% of FFP Type III injuries were treated using a 360° concept. For FFP Type IV injuries, the isolated posterior stabilization was the predominant operative method (80.4%), and 360° stabilization was applied in only 19.0% of the operated cases.

## 4. Discussion

This study represents the largest analysis of fragility fractures of the pelvic ring (FFPs) in Germany to date, encompassing 1242 patients. The selection of patients adhered strictly to the criteria originally established by Rommens et al. (geriatric patients with low energy/non-existing trauma) [3], ensuring comparability with existing research. The distribution of fracture types in our cohort closely aligns with previous studies, reaffirming the validity of the FFP classification system [11]. FFP Type II fractures were the most prevalent, followed by Types IV, I, and III. In contrast, therapeutic strategies, especially for Type III and Type IV fractures, where a more aggressive surgical approach used to be recommended [12], appear to have shifted and advanced over the past decade, with a greater emphasis on minimally invasive procedures compared to earlier recommendations [7].

### 4.1. Therapy by Classification: Operative or Non-Operative?

The literature presents varying approaches to the treatment of FFPs, with therapeutic decisions heavily influenced by fracture classification [4,13,14,15]. Rommens et al. suggested that surgical intervention is generally unnecessary for FFP Type I [4]. This aligns with the findings in our cohort, where only 7 out of 258 (2.7%) patients with FFP Type I underwent surgical stabilization. The primary reasons for surgery in these cases were likely persistent pain and prolonged immobility caused by the injury. Clinical experience indicates that operative stabilization may occasionally be necessary in such cases. Furthermore, our clinical experience indicates that not only the type of fracture but also the activity and progress of osteoporosis and bone waste are factors influencing stabilization methods. It is also important to note that fractures initially managed conservatively with rapid progression may exhibit instability over time, as highlighted by Rommens et al. in their observational study [16]. While such progression would warrant reclassification, these changes may not always be captured in registry data. Another frequent reason for delayed surgical intervention is the development of non-unions in the anterior pelvic ring, which has been identified as a significant adverse outcome in elderly patients [3].

For FFP Type II fractures, posterior pelvic ring instability is an additional concern. Conservative management with pain medication, osteoanabolic treatment, and early mobilization may lead to an “upgrade” of FFPs, as noted by Rommens et al. [4,13,14,16]. Consequently, the authors recommend considering surgical stabilization for these injuries. In our cohort, 20.9% of patients with FFP Type II fractures underwent surgical stabilization, while the majority were treated non-operatively. When examining the subcategories of FFP Type II, it becomes evident that surgical intervention rates increase with the severity of posterior instability: Type IIa (15%), Type IIb (19%), and Type IIc (29%).

However, it remains unclear whether the decision for surgical intervention was driven primarily by the fracture morphology and FFP classification seen on initial CT imaging or by clinical symptoms (e.g., pain, immobilization) indicating higher levels of instability in the posterior ring.

According to Rommens et al. [3], injuries classified as FFP Type III typically require open stabilization. In contrast, in our cohort, open stabilization was only performed in 49% of iliac fractures, 7% of sacroiliac (SI) joint fractures, and 43% of sacral fractures. This might be due to significantly lower complication rates of percutaneous operations [17]. In addition, Rommens himself noted in a more recent article [18] that percutaneous techniques are emerging and are gaining more interest and importance, as supported by the rising numbers of publications on this subject over recent years.

Among FFP Type IV injuries, spinopelvic dissociation (subtype IVb) warrants particular attention. Due to the severe instability of the posterior pelvic ring, spinopelvic fixation is often recommended for maximum mechanical stability, as also described by Rommens et al. [4]. In our cohort, surgical treatment was specified for 138 patients that suffered a Type IVb injury. Spinopelvic fixation was performed in only 16 of these patients (12%). Interestingly, far more patients with the same injury pattern (n = 68, 49%) were treated using closed reduction and stabilization with percutaneous screws. These findings are consistent with those reported by Wilson et al. [8] who reviewed 766 patients with FFPs in 2021. Among them, 463 patients underwent surgical stabilization, primarily of the posterior pelvic ring, with spinopelvic fixation used in only 1% of cases. Similarly, Heiman et al. [9] highlighted the limited evaluation of spinopelvic fixation techniques in the literature, emphasizing the need for further research [19,20]. Wilson et al. (2021) [8] concluded that spinopelvic fixation should be reserved for the most unstable and complex fracture patterns, where less invasive stabilization methods are insufficient.

In summary, significant heterogeneity exists in the literature regarding indications for surgical intervention and stabilization techniques for FFPs. A recent survey among pelvic surgery experts [21] highlighted the urgent need to establish comprehensive guidelines for the diagnosis and treatment of FFPs.

### 4.2. Stabilization Methods: Isolated Anterior, Isolated Posterior, or 360° Stabilization?

Isolated anterior pelvic ring stabilization appears obsolete, as fragility fractures in this region do not cause instability. As pelvic ring instability increases, corresponding to higher classifications in the FFP system, treatment tends to focus more on stabilizing the posterior pelvic ring. In our cohort, isolated posterior ring stabilization was performed in 50% of operated FFP Type II and FFP Type III fractures. Combined stabilization of both the anterior and posterior pelvic ring occurred at nearly the same frequency: 48% for FFP Type II and 50.0% for FFP Type III fractures. For FFP Type IV fractures, isolated stabilization of the posterior pelvic ring was performed in 80.5% of cases, while combined stabilization was used in only 19.0% of cases.

The concept of 360° stabilization for geriatric pelvic ring fractures is frequently cited in the literature [22,23,24]. This approach assumes that an insufficiency fracture of the posterior ring combined with anterior pelvic ring disruption requires absolute stability to ensure proper healing, at least temporarily. However, this contrasts with the concept of isolated dorsal stabilization, which was applied equally to FFP Type II and FFP Type III fractures in our cohort. The literature often highlights the relatively minor role of anterior ring stability in maintaining overall pelvic ring integrity, supporting dorsal stabilization as a standalone technique [25]. Numerous studies have demonstrated the safety and comparable outcomes of this approach, suggesting that isolated dorsal stabilization may suffice in most cases [26,27]. The current data highlight the ongoing disagreement regarding the optimal therapeutic approach for FFPs [28,29] and furthermore highlight the serious character of these injuries, especially in the geriatric population [30]. In addition, different treatment strategies may also vary between different levels of trauma centers. Especially, the use of more advanced technologies like 3D navigation or angular stable implants for the stabilization of the posterior pelvic ring, which are considered to render additional anterior stabilization unnecessary, continue to be predominantly limited to high-resource centers.

### 4.3. Techniques for Stabilizing the Anterior and Posterior Pelvic Ring

#### 4.3.1. Anterior Pelvic Ring

The supraacetabular external fixator has traditionally played a significant role as a standard method for emergency and definitive mechanical stabilization of the pelvic ring, particularly in high-energy trauma cases [31,32,33]. In the geriatric population, the external fixator has also shown effectiveness, particularly in controlling pain associated with fractures [34]. However, its role in treating FFPs has become controversial, partly due to complications reported in some studies [35]. This shift is reflected in our data, where external fixation was used only in 33 cases, compared to 75 cases treated with percutaneous screw osteosynthesis, which was the most frequently employed method for anterior stabilization in our cohort. This also aligns with the literature [36].

Open reduction and plate osteosynthesis, although used less frequently (15.1%), deserve mention. This technique is often associated with complications such as screw loosening, particularly near the symphysis [37]. To mitigate these issues, some authors recommend the use of dual plates, which increases invasiveness [33]. This approach, however, warrants critical consideration given the generally minor role of anterior ring stabilization in maintaining overall pelvic stability.

#### 4.3.2. Posterior Pelvic Ring

Screw osteosynthesis was the most frequently used technique for stabilizing the posterior pelvic ring, applied in 42% of cases involving the ilium, 66% for the SI joints, and 56% for the sacrum.

Closed reduction and percutaneous screw osteosynthesis for the SI joints and sacrum have been shown in multiple studies to be safe, with low complication rates and favorable outcomes [38]. Additionally, 3D navigation-assisted implantation of SI screws has demonstrated reliable safety and effectiveness [39,40].

However, loosening of SI screws is a commonly reported complication, often attributed to reduced bone mass in the S1 and S2 corridors [41]. Kim et al. [42], in a study involving 110 patients, identified screw loosening particularly in pelvic ring injuries with a translational component.

In our cohort, screw osteosynthesis was used in 66% of SI joint stabilizations and in 56% of sacral stabilizations via percutaneous screws. Open surgical approaches, such as open reduction and internal fixation with SI screws or spinopelvic fixation, were performed less frequently (7% and 43%, respectively).

Regarding the use of navigation for percutaneous screw placement in the posterior pelvic ring, our cohort demonstrates that this technique is already well established, with 84 of 187 screws (45%) being placed under navigation. The literature also supports the safety of navigated sacroiliac (SI) screw placement [39,43]. Additionally, navigation has been shown to be superior to conventional techniques, such as two-dimensional fluoroscopy, in certain settings, particularly for complex anatomical cases like dysmorphic sacra [44].

The spinopelvic fixator is a recognized option for stabilizing the posterior pelvic ring [45], particularly in spinopelvic dissociation (FFP Type IVb), and was used in 34% of sacral stabilization cases in our cohort. Closed reduction with percutaneous screw osteosynthesis was applied at a slightly higher frequency (56%). Less invasive techniques, such as bilateral vertebropelvic stabilization, have also been introduced [46].

## 5. Limitations

This study is subject to several limitations inherent to registry-based research. First, the retrospective nature of the data collection relies on the accuracy and completeness of the information entered by participating centers, which may introduce reporting bias or data inconsistencies. Second, the classification and therapeutic decisions documented may vary across institutions, reflecting differences in clinical practices and expertise rather than standardized protocols. Third, the lack of longitudinal follow-up in the registry limits the ability to assess long-term outcomes, such as functional recovery or delayed complications like pseudarthrosis. Finally, some specific procedural details or emerging techniques may not have been captured due to the limitations of the registry framework. These factors highlight the need for prospective, multicenter studies to validate and refine the findings presented here.

## 6. Conclusions

This study represents a snapshot throughout 22 trauma centers in Germany and Austria, allowing for evaluation of the state of the art in epidemiology and treatment of fragility fractures of the pelvic ring (FFPs) in Germany, highlighting the prevalence of FFP Type II fractures and the variability in treatment strategies based on fracture classification. While non-operative management dominates, surgical interventions are increasingly used for higher-grade fractures, supported by advancements in minimally invasive techniques.

The findings emphasize the further need for standardized, evidence-based guidelines and further research to optimize therapeutic approaches and evaluate long-term outcomes. Collaborative efforts remain essential to improve care for geriatric patients with FFPs.

## Figures and Tables

**Table 1:** Distribution of patients by classification (FFP).

Classification	Total n (%)
**FFP Type I**	**258 (20.8)**
Type Ia	243 (19.6)
Type Ib	15 (1.2)
**FFP Type II**	**631 (50.8)**
Type IIa	72 (5.8)
Type IIb	394 (31.7)
Type IIc	165 (13.3)
**FFP Type III**	**91 (7.3)**
Type IIIa	34 (2.7)
Type IIIb	13 (1.0)
Type IIIc	44 (3.5)
**FFP Type IV**	**262 (21.1)**
Type IVa	16 (1.3)
Type IVb	191 (15.4)
Type IVc	55 (4.4)
**TOTAL**	**1242 (100)**

**Table 2:** Implants used for stabilization of the pelvic ring.

	FFP Classification *n*				
Implants Used	I	II	III	IV	TOTAL *n* (%)
**Os pubis**	**5**	**67**	**28**	**39**	**139 (100)**
Plate	1	7	8	5	21 (15.1)
External Fixator	1	13	6	13	33 (23.7)
Internal Fixator	0	5	1	4	10 (7.2)
Screw total	3	42	13	17	75 (54.0)
Screw	3	37	13	15	68 (90.7 *)
Screw navigated	0	5	0	2	7 (9.3 *)
**Ilium**	**1**	**0**	**25**	**7**	**33 (100)**
Plate	0	0	10	1	11 (33.3)
Plate and screw	1	0	4	0	5 (15.2)
Screw total	0	0	9	5	14 (42.4)
Screw	0	0	7	5	12 (86.0 *)
Screw navigated	0	0	2	0	2 (14.0 *)
Other **	0	0	2	1	3 (9.1)
**SI joint**	**0**	**86**	**22**	**101**	**209 (100)**
Screw total	0	53	17	68	138 (66.0)
Screw	0	29	13	49	91 (66.0 *)
Screw navigated	0	24	4	19	47 (34.0 *)
Spinopelvic fixation	0	0	1	6	7 (3.5)
ORIF other	0	3	2	2	7 (3.5)
Other **	0	30	2	25	57 (27.0)
**Sacrum**	**0**	**18**	**8**	**36**	**62 (100)**
Screw navigated	0	11	5	19	35 (56.0)
Spinopelvic fixation	0	3	3	15	21 (34.0)
ORIF other **	0	4	0	2	6 (10.0)

* % of total screws (navigated and non-navigated). ** Although the registry allows specifying implants for each case, responses marked as “other” were included in the relative data calculations. Unanswered implant choices were excluded from the calculation of valid percentages.

**Table 3:** Treatment according to FFP classification.

FFP Type *n* (%)	I	II	III	IV
Total	258	631	91	262
Non-operative	251 (97.3)	497 (79.0)	35 (38.5)	71 (27.0)
Operative	7 (2.7)	132 (21.0)	56 (61.5)	189 (73.0)
Anterior only	5 (71.4 *)	2 (1.5 *)	0	1 (0.5 *)
Posterior only	2 (28.6 *)	66 (50.0 *)	28 (50.0 *)	152 (80.5 *)
Combined (360°)	0	63 (47.7 *)	28 (50.0 *)	36 (19.0 *)
Not specified	0	1 (0.8 *)	0	0

* % of operatively treated patients in the respective FFP subgroup.

## Data Availability

The datasets generated and analyzed during the current study are available from the corresponding author upon reasonable request.

## Abbreviations

The following abbreviations are used in this manuscript:

FFP	Fragility fracture of the pelvic ring
DGU	Deutsche Gesellschaft für Unfallchirurgie (German Traumatology Society)
GDPR	General Data Protection Regulation
IQR	Inter Quartile Range
ASA	American Society of Anesthesiologists
SI	Sacroiliac
ORIF	Open reduction and internal fixation
